# Notice, Respecting an Artificial Stony Substance

**Published:** 1810-10

**Authors:** F. R. Cureaudeau


### *Notice, respecting an Artificial Stony Substance.*

By F. R. Cureaudeau.

*From the Journal de Physique.*

A REMARKABLE example of the great degree of solidity which water may acquire in certain combinations, is that which artificial stones present to us.

The stones into the composition of which water enters and forms more than half the weight, are composed of one part of acid and of sulphur, and of two parts of baked clay, reduced to powder.

Simply mixing these three substances does not give a solution of sulphate of clay, but it favours the reciprocal action of these three substances on each other, and produces much igneous fluid, the emission of which is sometimes so considerable that the matter appears to be even at a white heat.

This beautiful phenomenon continues more than one hour, if it be operated on by a mixture of 25 or 30 quintals; but what is the most remarkable is, that if sufficient water is not added at first, and the remainder afterwards added, the moment the defect appears, and the re-action of the substances on one another be the most energetic, then the mass becomes again a liquid; but it acquires all at once a great degree of solidity; the heat heat which it produces is even augmented to a high intensity, and the matter passes almost to a state of insolubility.

This last property, by which a mixture usually producing soluble salts, shews the penetration and union of water and acid with the earth to be so great, as to form a stony substance, possessing all the properties of stones except insolubility.

I have considered of what importance a factitious stony substance of these particular properties can be made. Could it not be employed with much advantage to form cements to join statues, to model vases, and many other purposes which experience will shew? It is true that it is necessary to preserve the bodies that are to compose this artificial stony cement from the influence of water and moisture.

Another consideration which makes me think this composition will appear with some interest, is the theory of its formation, and its analogy with the stones of solfaterra, and inclines me to have recourse to the hypotheses of subterraneous fires mixed with combustible matter, to explain volcanic eruptions. In effect, since pure water passing instantaneously from a liquid to a solid state, may produce the development of considerable heat, may it not be the immediate cause of volcanic eruptions?

